# Resveratrol inhibits LPS-induced apoptosis in VSC4.1 motoneurons through enhancing SIRT1-mediated autophagy 

**DOI:** 10.22038/ijbms.2020.44534.10416

**Published:** 2021-01

**Authors:** He Tian, Haosen Zhao, Xifan Mei, Daoyong Li, Jiaquan Lin, Sen Lin, Changwei Song

**Affiliations:** 1 Department of Histology and Embryology, School of Basic Medicine, Jinzhou Medical University, Liaoning 121000, China; 2 Department of Orthopedics, First Affiliated Hospital of Jinzhou Medical University, Liaoning 121000, China; 3 Medical College of Jinzhou Medical University, Liaoning 121000, China

**Keywords:** Apoptosis, Autophagy, Lipopolysaccharide, Resveratrol, Sirtuin 1, Spinal cord injury

## Abstract

**Objective(s):**

Resveratrol has been recognized as a potential therapeutic drug in spinal cord injury (SCI). Sirtuin 1 (SIRT1) is vital in the regulation of apoptosis and cell stress response. In this research, our purpose was to explore the mechanisms of resveratrol on neuroprotection and to explore the role of SIRT1.

**Materials and Methods:**

We used lipopolysaccharide (LPS) in the VSC4.1 spinal cord neuron cell line to mimic the micro-environment of the injured spinal cord. The apoptosis of VSC4.1 motoneurons was assessed by TUNEL staining, Western blot, and RT-PCR. Immunofluorescence staining was used to observe the expression site of SIRT1, LC3-B, and Beclin-1, and their protein levels were measured by Western blot and RT-PCR.

**Results:**

Our results showed that resveratrol inhibits LPS-induced apoptosis in VSC4.1 motoneurons. Levels of LC3-B, beclin-1, and SIRT1 indicated a significant increase after resveratrol treatment. But, if autophagy was inhibited, apoptosis in VSC4.1 motoneurons significantly increased. When the cells were treated with EX527, a SIRT1 inhibitor, the protein contents of LC3-B and Beclin-1 were suppressed.

**Conclusion:**

Resveratrol inhibits apoptosis through promoting autophagy in VSC4.1 motoneurons. SIRT1 was involved in autophagy activated by resveratrol in VSC4.1 motoneurons.

## Introduction

Spinal cord injury (SCI), which leads to serious nervous system damage, is one of the main causes of disability. After SCI, a lot of neuronal apoptosis, especially of motor neurons, occurs during the secondary injury process (1). The secondary injury process results in numerous neuronal losses, which are important mechanisms of secondary injury (2, 3). Thus, inhibiting neuronal apoptosis and reducing secondary injury are among the treatment strategies for SCI (4). 

Autophagy is a primary means of intracellular degradation of proteins and organelles in the cytoplasm (5). Autophagy function abnormalities can cause various diseases and autophagic cell death (6, 7). However, more and more studies indicate that autophagy functions as one of the protective factors in numerous diseases and serves a considerable role in neurodegenerative diseases (8).

Sirtuin 1 (SIRT1) is a deacetylase dependent on nicotinamide adenosine dinucleotide (NAD). It is a class III histone deacetylase belonging to the sirtuin family. SIRT1 is vital in regulating the cell stress response, apoptosis, metabolism, and survival (9-11). Recent studies show that up-regulation of SIRT1 can be a therapeutic target for neurodegeneration diseases (12, 13). Studies showed that after up-regulation of SIRT1, autophagy expression increases significantly in some diseases (14, 15). However, the possible therapeutic effect of SIRT1 activation for SCI and the potential relevance of SIRT1, autophagy after SCI is still not clear.

In this research, we used LPS to treat VSC4.1 motor neuron cell line to mimic the micro-environment of an injured spinal cord. LPS is the main component of the outer membrane of gram-negative bacteria, and it is widely used to induce inflammatory responses (16). LPS increased the expression of inflammatory factors, such as TNF-α in macrophages, epithelial cells, astrocytes, and microglia (17-19). It has been confirmed that acute LPS application induced inflammatory responses and apoptosis of neurons in the brain and spinal cord (20-22). We also used resveratrol to up-regulate SIRT1 expression. 3-MA and EX527 were used to inhibit autophagy and expression of SIRT1. EX527 is a potent and selective SIRT1 inhibitor that can effectively inhibit SIRT1 deacetylase activity. 3-MA blocks autophagy through the action of phosphoinositide 3-phosphate kinase (PI3K). 

Our results showed that resveratrol protects VSC4.1 motoneurons against LPS- induced apoptosis. Resveratrol promoted autophagy and up-regulated SIRT1. Autophagy inhibition increased apoptosis, however, expression of SIRT1 demonstrated no significant difference. Interestingly, autophagy decreased when SIRT1 was inhibited, but apoptosis increased significantly. All these findings indicated that SIRT1 up-regulation played a significant role in the protection of resveratrol against apoptosis by promoting autophagy after SCI. 

## Materials and Methods

***Cell culture***

VSC4.1 cells are a spinal cord anterior horn motor neuron tumor cell line. VSC4.1 motoneurons were cultured in high glucose Dulbecco’s modified eagle medium (DMEM) (Hyclone, Utah, USA). 15% (v/v) FBS (Gibco, USA) and 1% (w/v) penicillin and streptomycin (Gibco, USA) were added to the medium. The cells were stored at 37 °C in an incubator for subsequent use.

**Cell viability assay**

3-(4, 5-dimethylthiazol-2-yl) -2,5-diphenyltetrazolium bromide (MTT) was used to detected cell survival rate. In order to screen the appropriate concentration of resveratrol, MTT assay was used. Incubated in a thermostatic incubator at 37 °C for 24 hr with resveratrol concentration gradient (0, 2, 4, 6, 8, 10, 12, 15, 18, and 20 μM), MTT (20 μl, 5 μg/ml, Sigma, Germany) was added to the medium in each well. Next, the media were dumped and a 150 μl dimethyl sulfoxide (DMSO, Sigma, Germany) was added. Then, the VSC4.1 cells were incubated for 4 hr at 37 °C. A spectrophotometer (Thermo Fisher Scientific, Massachusetts) was used to detect absorbance at 490 nm; 5 wells were calculated per condition.

***Cell treatment***

Cells were divided into five groups, the names of groups were control, LPS, resveratrol, EX527, and 3-MA. The cells of the control group were cultured in the medium only. The other four groups were all treated with lipopolysaccharide (16-19) (LPS, 10 μg/ml, Sigma, Darmstadt, Germany). Resveratrol (10 μM, Dalianmeilun, Dalian, China) was added to the resveratrol group. Resveratrol and 3-MA (10 mM, Selleck, Texas, USA) were added to the 3-MA group. Resveratrol and EX527 (20 μM, Selleck, Texas, USA) were added to the EX527 group. The cells were harvested and tested one day post-treatment.

***TUNEL staining***

Apoptosis was detected by TUNEL Apo-Green Death Detection Kit (Biotool, Texas, USA). Once washed, fixed, and blocked, as previously described, the cells were incubated with the TUNEL reaction mixture at 37 °C for 1 hr, and the nuclei were counterstained with the DAPI solution (1:1000) at room temperature for 15 min. The analysis was conducted using a fluorescence microscope and each group was repeated three times to perform statistical analysis.

***Immunofluorescence analysis***

The cells were washed twice with phosphate buffer solution (PBS) buffer, then 4% paraformaldehyde was added in order to fix the cells. After fixing for 10 min, the cells were incubated with goat serum (ZSGB-Bio, China) at 4 °C for 2 hr. Cell incubation in the primary antibodies exceeds 12 hr. The primary antibodies include rabbit anti-SIRT1 (1:200, Cell Signalling, USA) and rabbit anti-LC3-B (1:200, Abcam, USA). The next day, after washing with PBS three times, the cells were incubated with mouse anti-β-Tubulin at room temperature for 2 hr. After washing again, the cells were incubated with a mixture composed of goat anti-rabbit IgG (1:500; Bioss, China) and goat anti-mouse IgG (1:500; Bioss, China) at room temperature for 2 hr. Finally, the DAPI solution (1:1000) was used to dye the nucleus. Fluorescence microscopy was used for the observation.

***Western blot analysis***

When the cells were treated for 24 hr, the cells were dissolved in RIPA lysis buffer (Beyotime, China) after being collected and washed with 1×PBS twice. The final protein concentration (2 μg/μl) was quantitated by the BCA kit (EnoGene, Nanjing, China). The prepared samples (40 μg) were added into different lanes. The protein samples were transferred onto polyvinylidene fluoride membranes after electrophoresis in SDS-PAGE gels of 12% concentration. 5% skimmed milk Tris-buffered saline with Tween 20 (TBST) solution was used to reduce or eliminate non-specific binding protein molecules on the carrier. The membranes were blocked at room temperature for 2 hr. Then, primary antibodies were added respectively, the membranes were incubated with primary antibodies for 16 hr at 4 °C. The antibody dilution concentrations were anti-SIRT1 (1:1000, Cell Signalling), anti-Cleaved Caspase-3 (1:1000, Novus, USA), anti-Caspase-9 (1:1000, Novus, USA), anti-Caspase-8 (1:500, Beyotime Biotechnology, China), anti-LC3-B (1:1000, Abcam, USA), anti-Beclin-1 (1:500, Novus, USA), and anti-β-actin (1:1000, Abcam, USA). On the second day, after washing with TBST three times, the membranes were incubated with secondary antibodies (1:10,000, Earthox, USA) at room temperature for 2 hr. Then, ECL was used for color development. Western blot bands were observed by Automated Chemiluminescence Image Analysis System (Tanon, China). 

***RT-PCR analysis***

Trizol reagent (Invitrogen, Massachusetts, USA) was used to extract the total RNA of cells after treatment. RNA PCR Kit (Takara, Dalian, China) was utilized to synthesize cDNA. RT-PCR amplification was conducted using cDNA as the transcription template with the following primers:

SIRT1 (forward primer 5’-ATGATTGGCACCGATCCTCG-3’ and reverse primer 5’-ATTCCTGCAACCTGCTCCAA-3’); Beclin-1 (forward primer 5’-AAAGAGTGGAAGATGTCCGGC-3’ and reverse primer 5’-CAGCTGCTTCTCACCCTTGTA-3’); 

Caspase 3 (forward primer 5’-TGGCGATGAACTGGACAACA-3’ and reverse primer 5’-TAGAAAAGGGCAACCACCCG-3’); β-actin (forward primer 5’- ATATCGCTGCGCTCGTCG-3’ and reverse primer 5’-CAATGCCGTGTTC AATGGGG-3’). Reaction conditions are as follows: 5 min at 90 °C, followed by 27 cycles of 30 sec at 90 °C, 30 sec at 60 °C, 30 sec at 70 °C, and a final extension at 70 °C for 10 min.

***Statistical analysis***

Experimental data was represented in mean±SD and analyzed with SPSS 22.0. Unpaired Student’s t-test and one-way ANOVA were respectively used for comparison between two groups or among a plurality of groups. When using ANOVA, *post hoc* test was carried out by Student–Newman–Keuls test. *P*<0.05 was considered statistically significant.

## Results

***Resveratrol inhibits LPS-induced apoptosis in VSC4.1 motoneurons***

**Figure 1:**
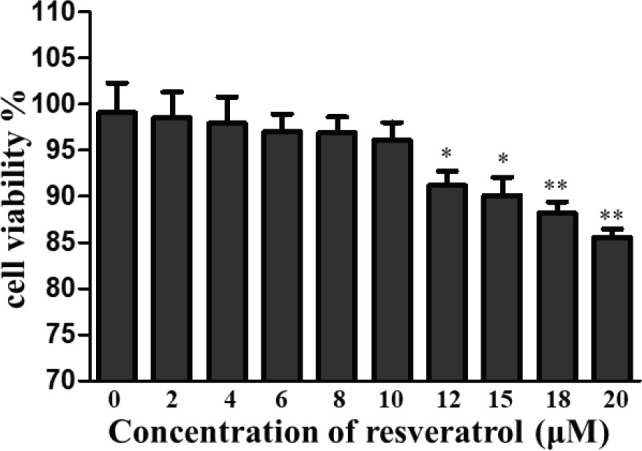
MTT assay results showed no significant injurious effects due to resveratrol treatment from 2 to 10 μM concentrations. *P<0.05, **P<0.01 in comparison with control group. n=5 for each group

In order to choose the suitable concentration of resveratrol, the MTT assay was used to detect cell viability of VSC4.1 motoneuronals. VSC4.1 anterior horn motor neurons were incubated with resveratrol concentrations at 0, 2, 4, 6, 8, 10, 12, 15, 18, and 20 μM for 24 hr. According to our results, the maximum concentration of resveratrol showing no significant injurious effects on cells was 10 μM (Figure 1). Thus, 10 μM was selected as the resveratrol concentration in this experiment. 

**Figure 2:**
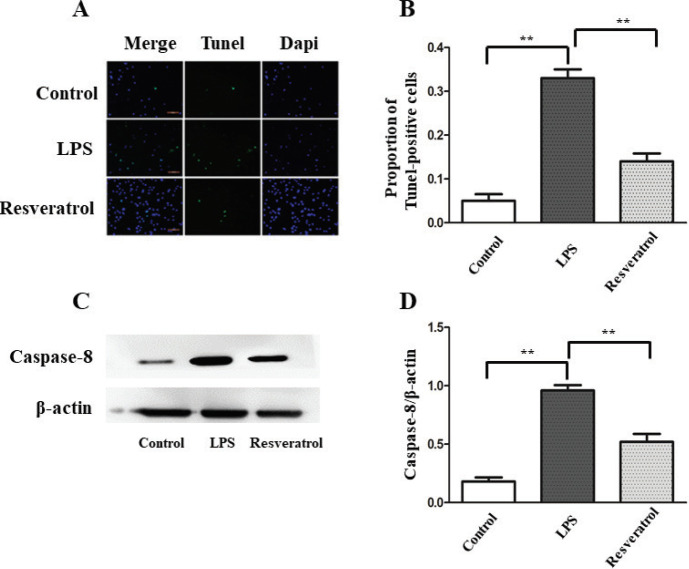
(A) Cell apoptosis was detected by TUNEL staining. (B) In the comparison between LPS and control groups, the number of TUNEL-positive cells showed a significant increase. On the contrary, resveratrol treatment reduced the proportion of apoptotic cells compared with the LPS group. The scale bar stands for 100 μm. **P<0.01 indicates significant differences between groups, n=4 for each group. (C, D) Comparing with control, Caspase-8 protein level increased obviously after LPS stimulation. Resveratrol inhibited the expression of Caspase-8, **P<0.01 indicates significant differences between groups, n=5 for each group

To test whether resveratrol modulated cellular apoptosis, TUNEL staining was performed. Results showed that LPS treatment increased apoptotic cells significantly, while treatment by resveratrol strongly reduced the amount of TUNEL positive cells (Figures 2A, B). Results from Western blot also confirmed that resveratrol inhibited LPS-induced apoptosis in VSC4.1 motoneuronals. As shown in Figure 2 (C, D) and Figure 5 (A, B, C), the protein levels of Caspase-8, Cleaved Caspase-3, and Caspase-9 in the resveratrol group were significantly down-regulated compared with the LPS-treated group. The mRNA expression of Caspase-3 detected by RT-PCR showed a similar result (Figures 5 D, E).

***Resveratrol inhibits apoptosis by promoting autophagy in VSC4.1 motoneurons***

**Figure 3:**
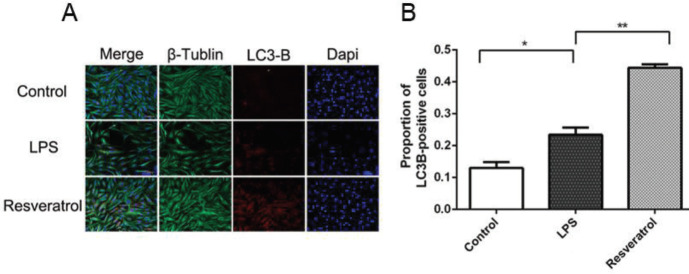
(A) Immunofluorescence analysis was used to observe the expression of LC3-B. (B) LC3-B exhibited a significant increase in the LPS group compared with control cells. Furthermore, resveratrol increased the proportion of LC3-B positive cells compared with LPS treatment. *P<0.05, **P<0.01 in comparison between groups, n=4 for each group

Immunofluorescence analysis revealed that LC3-B was up-regulated in VSC4.1 motoneuronals treated with LPS, and resveratrol administration increased LC3-B expression more significantly (Figure 3). Western blot and RT-PCR results showed that expression of beclin-1 and LC3-B obviously increased with resveratrol in comparison with the LPS group (Figure 6). In order to explore the relationship between apoptosis and autophagy, 3-MA, the autophagy inhibitor was used. As shown in Figure 5, resveratrol reduced apoptosis, but if autophagy was suppressed, the levels of cleaved caspase-3 and caspase-9 raised significantly. Expression of caspase-3 mRNA was detected by RT-PCR showed a similar result. These findings demonstrated that resveratrol inhibits apoptosis through promoting autophagy in VSC4.1 motoneurons.

***SIRT1 was involved in autophagy activated by resveratrol in VSC4.1 motoneurons***

**Figure 4:**
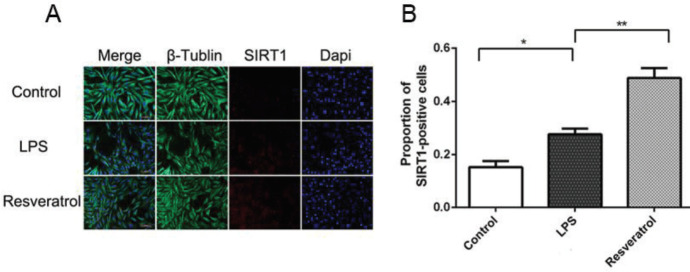
(A) Variation of SIRT1 was detected by immunofluorescence. (B) LPS could increase the expression of SIRT1, and resveratrol could raise the expression of SIRT1 further. In comparison between groups, *P<0.05, **P<0.01 indicates a significant difference, n=4 for each group

SIRT1: Sirtuin 1; LPS: lipopolysaccharide

**Figure 5:**
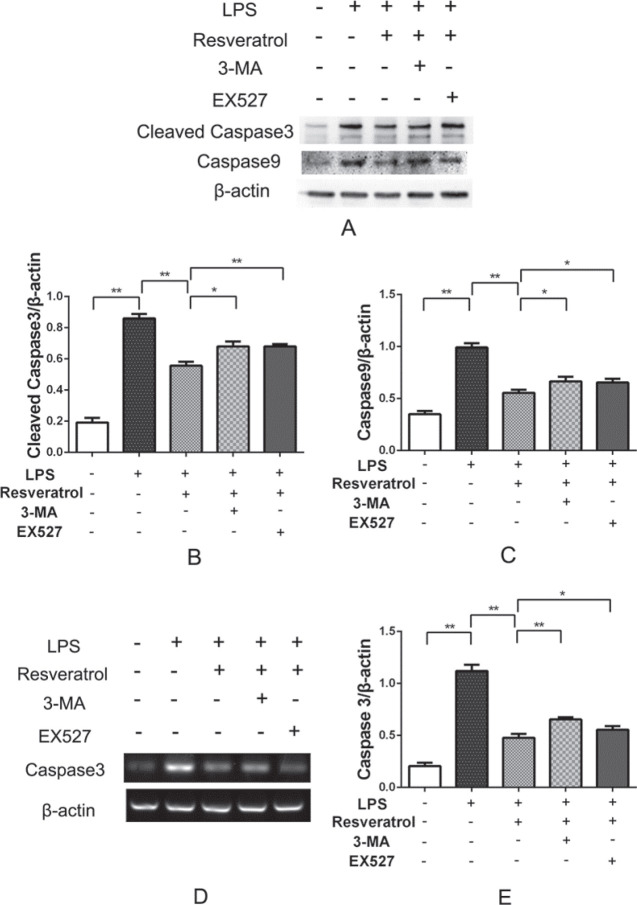
Protein and mRNA expressions of Caspase-3 and Caspase-9 were detected using Western blot (A) and RT-PCR (D). (B) and (C) respectively showed the average relative gray of Cleaved Caspase-3 and Caspase-9 protein expressions compared with the β-actin protein. (E) shows the average relative gray of Caspase-9 mRNA expression. *P<0.05, **P<0.01 in comparison between groups, n=4 for each group

**Figure 6:**
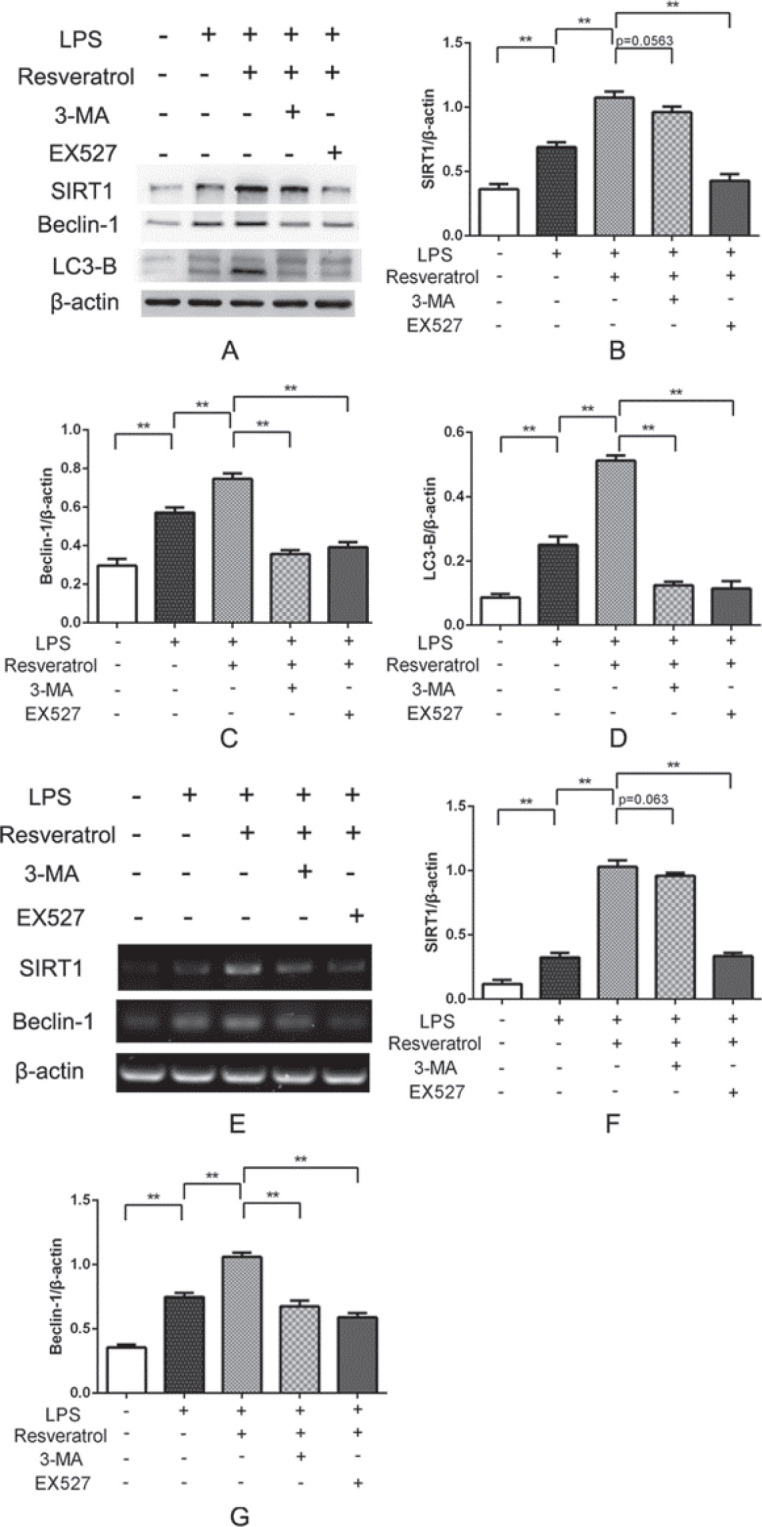
Expressions of SIRT1, Beclin-1, and LC3-B were examined using Western blot (A) and RT-PCR (E). The average relative gray of SIRT1, Beclin-1, and LC3-B compared with β-actin protein was displayed respectively in (B), (C), and (D). (F) and (G) show mRNA expressions of SIRT1 and Beclin-1, respectively. *P<0.05, **P<0.01, compared between groups, n=4 for each group

The results from immunofluorescence double-staining revealed that SIRT1 was up-regulated in VSC4.1 motoneuronals post-LPS treatment, and resveratrol increased SIRT1 significantly (Figure 4). To observe the function of SIRT1 in autophagy, EX527 was used to suppress SIRT1. Western blot and RT-PCR results revealed that the level of SIRT1 in the cells treated with resveratrol showed significant up-regulation, whereas it was evidently down-regulated with treatment by EX527, the SIRT1 inhibitor (Figure 6). The levels of beclin-1 and LC3-B evidently decreased in the EX527 group (Figure 6). RT-PCR results were consistent with those of the Western blot (Figure 6). These results indicate that SIRT1 suppression could reduce autophagy in VSC4.1 motoneurons after LPS treatment. However, there was no variation in the expression of SIRT1 when treated with 3-MA, the inhibitor of autophagy (Figure 6). But, if the cells were treated with EX527, a SIRT1 inhibitor, the protein levels of cleaved caspase-3 and caspase-9 increased significantly (Figure 5). RT-PCR showed a similar result (Figure 5). The experimental results indicate LPS-induced apoptosis in the VSC4.1 motoneurons increase if SIRT1 and autophagy were suppressed.

MTT: 3-(4, 5-dimethylthiazol-2-yl) -2,5-diphenyltetrazolium bromide

LPS: lipopolysaccharide

LPS: lipopolysaccharide

SIRT1: Sirtuin 1

## Discussion

Sirtuins are NAD +/- dependent deacetylases that play vital roles in metabolism and stress adaptations. SIRT1, a class III histone deacetylase, belongs to the sirtuin family and regulates cell stress response, apoptosis, metabolism, and survival. Numerous studies have confirmed the relationship between SIRT1 and a number of physiological functions, including apoptosis, inflammation, and aging (10, 23). Recent studies have indicated that SIRT1 is strongly implicated in neurodegenerative diseases, including Alzheimer’s disease as well as ischemic stroke and traumatic brain injury to the central nervous system (14, 24). Up-regulation of SIRT1 by resveratrol showed protective effects in diseases such as metabolic syndrome and neurodegenerative disorders (25, 26). In our research, resveratrol was used to up-regulate SIRT1. The SIRT1 expression showed a significant increase compared with the resveratrol and LPS-treated groups. Treatment with resveratrol also promoted the synthesis of autophagy proteins, such as Beclin-1 or LC3-B, indicating that the over-expression of SIRT1 may increase autophagy expression levels after SCI. By contrast, the levels of cleaved Caspase-3 and Caspase-9, as well as the number of TUNEL-positive cells, showed a remarkable decline after resveratrol treatment. This result revealed that SIRT1 up-regulation may reduce the expression level of apoptosis and may become neuroprotective. 

As a primary means for the intracellular degradation of proteins and organelles in the cytoplasm, autophagy showed low expressions in normal circumstances to eliminate damaged or long-lived proteins (27). When the environment stimuli change, the autophagy expression increases, which plays a crucial role in the pathological processes of numerous diseases, such as infections and neurodegenerative disorders (28, 29). In this research, the LPS-treated group exhibited obvious raise in Beclin-1 and LC3-B compared with the control group. Our findings are consistent with these studies. 

Studies demonstrated that SIRT1 up-regulation can promote the deacetylation of vital autophagic regulators such as ATG5 and ATG7, which suggests the relationship between SIRT1 and autophagy (30). In our study, we utilized EX527, a SIRT1 inhibitor, to restrain SIRT1 expression and to down-regulate autophagy using the autophagy inhibitor, 3-MA. In the EX527-treated group, the levels of SIRT1, Beclin-1, and LC3-B indicated significant up-regulation compared with the resveratrol group. Compared with the resveratrol-administrated group, the Beclin-1 and LC3-B expressions were obviously inhibited in the 3-MA-intervened group. However, no obvious difference was observed in SIRT1 expression between the two groups. These results revealed that SIRT1 regulation affects autophagy expression after SCI and not vice versa. 

Research indicated the potential link between autophagy and apoptosis (31, 32). Given the key role of Beclin-1 in the beginning of autophagy, caspase activation always inhibits the autophagic pathway (33,34). A series of cellular stress responses can induce autophagy and apoptosis at the same time (35). Cleaved Caspase-3 and Caspase-9, as well as apoptotic cells in the resveratrol group, were markedly down-regulated compared with the LPS-treated cells. By contrast, if the cells were treated with autophagy inhibitor or SIRT1 inhibitor, the expressions of Cleaved Caspase-3 and Caspase-9 were significantly up-regulated. These results indicated that inhibition of SIRT1 or autophagy can increase apoptosis in VSC4.1 motoneurons after LPS treatment. Furthermore, the up-regulation results of SIRT1 on apoptosis may be in connection with the changes in autophagy. 

SCI, which leads to serious nervous system damage, is one of the main causes of disability. The pathophysiology of SCI includes primary and secondary injuries. Primary injury, including mechanical compression, bleeding, and electrolyte balance disorders, is irreversible. Secondary injury mainly includes post-traumatic inflammation, local tissue necrosis and apoptosis, oxidative stress, and neuronal apoptosis and necrosis, among others (36-38). Research showed that apoptosis of motoneurons is one of the main obstacles in recovering motor functions after SCI (39, 40).

This research primarily probes the neuroprotective effect of SIRT1 and the potential relationship among SIRT1, autophagy, and apoptosis using LPS-treated VSC4.1 motoneurons to mimic the micro-environment of an injured spinal cord. The VSC4.1 cell line from the ventral spinal cord neurons can simulate SCI to some extent. Our results indicated that SIRT1 up-regulation may play a vital part in neuroprotection and may help protect against apoptosis by promoting autophagy after LPS treatment in VSC4.1 motoneurons. SCI may also follow the same mechanism. However, this study contains insufficient information for this conclusion. Resveratrol has been proven to have neuroprotective effects in numerous neurodegenerative diseases (41). At the same time, it may protect VSC4.1 motoneurons from apoptosis through other signal pathways. However, whether this mechanism works in animals still requires further verification.

## Conclusion

The results of this study indicated that resveratrol had obvious anti-apoptosis effects on LPS-induced motoneurons. The mechanism may be related to activation of autophagy, and SIRT1 was involved in autophagy activated by resveratrol.

## References

[1] Zhaohui C, Shuihua W (2020). Protective Effects of SIRT6 against inflammation, oxidative stress, and cell apoptosis in spinal cord injury. Inflammation.

[2] Crowe MJ, Bresnahan JC, Shuman SL, Masters JN, Beattie MS (1997). Apoptosis and delayed degeneration after spinal cord injury in rats and monkeys. Nat Med.

[3] Wei HY, Ma X (2014). Tamoxifen reduces infiltration of inflammatory cells, apoptosis and inhibits IKK/NF-kB pathway after spinal cord injury in rats. Neurol Sci.

[4] Zhang Q, Xiong Y, Li B, Deng GY, Fu WW, Cao BC (2021). Total flavonoids of hawthorn leaves promote motor function recovery via inhibition of apoptosis after spinal cord injury. Neural Regen Res.

[5] Wu C, Xu H, Li J, Hu X, Wang X, Huang Y (2020). Baicalein Attenuates pyroptosis and endoplasmic reticulum stress following spinal cord ischemia-reperfusion injury via autophagy enhancement. Front Pharmacol.

[6] Guo H, He Y, Bu C, Peng Z (2019). Antitumor and apoptotic effects of 5-methoxypsoralen in U87MG human glioma cells and its effect on cell cycle, autophagy and PI3K/Akt signaling pathway. Arch Med Sci.

[7] Zhou K, Zheng Z, Li Y, Han W, Zhang J, Mao Y (2020). TFE3, a potential therapeutic target for spinal cord injury via augmenting autophagy flux and alleviating ER stress. Theranostics.

[8] Luo F, Sandhu AF, Rungratanawanich W, Williams GE, Akbar M, Zhou S (2020). Melatonin and autophagy in aging-related neurodegenerative diseases. Int J Mol Sci.

[9] Iside C, Scafuro M, Nebbioso A, Altucci L (2020). SIRT1 activation by natural phytochemicals: an overview. Front Pharmacol.

[10] Xu F, Xu J, Xiong X, Deng Y (2019). Salidroside inhibits MAPK, NF-κB, and STAT3 pathways in psoriasis-associated oxidative stress via SIRT1 activation. Redox Rep.

[11] Herranz D, Muñoz-Martin M, Cañamero M, Mulero F, Martinez-Pastor B, Fernandez-Capetillo O (2010). Sirt1 improves healthy ageing and protects from metabolic syndrome-associated cancer. Nat Commun.

[12] Stoyas CA, Bushart DD, Switonski PM, Ward JM, Alaghatta A, Tang MB (2020). Nicotinamide pathway-dependent sirt1 activation restores calcium homeostasis to achieve neuroprotection in spinocerebellar ataxia type 7. Neuron.

[13] Kreiner G, Sönmez A, Liss B, Parlato R (2019). Integration of the deacetylase SIRT1 in the response to nucleolar stress: metabolic implications for neurodegenerative diseases. Front Mol Neurosci.

[14] Cattelan A, Ceolotto G, Bova S, Albiero M, Kuppusamy M, De Martin S (2015). Nad+/- dependent sirt1 deactivation has a key role on ischemia–reperfusion-induced apoptosis. Vascul Pharmacol.

[15] Tian Q, Fan X, Ma J, Han Y, Li D, Jiang S (2020). Resveratrol ameliorates lipopolysaccharide-induced anxiety-like behavior by attenuating YAP-mediated neuro-inflammation and promoting hippocampal autophagy in mice. Toxicol Appl Pharmacol.

[16] Fei Gao, Zhiqiang Liu, Wei Ren, Wen Jiang (2014). Acute lipopolysaccharide exposure facilitates epileptiform activity via enhanced excitatory synaptic transmission and neuronal excitability in vitro. Neuropsychiatr Dis Treat.

[17] Derek S (2011). Wheeler, John S. Giuliano Jr, Patrick M. Lahni, Alvin Denenberg, Hector R. Wong, Basilia Zingarelli. The immunomodulatory effects of albumin and in vivo. Adv Pharmacol Sci.

[18] Shahid Husain, Gregory I Liou, Craig E Crosson (2011). Opioid receptor activation: suppression of ischemia/reperfusion-induced production of tnf-αin the retina. Invest Ophthalmol Vis Sci.

[19] Svetlana Nikolaeva, Vera Bachteeva, Ekaterina Fock, Sabine Herterich, Elena Lavrova, Alexandra Borodkina (2012). Frog urinary bladder epithelial cells express TLR4 and respond to bacterial LPS by increase of iNOS expression and L-arginine uptake. Am J Physiol Regul Integr Comp Physiol.

[20] Lir-Wan Fan, Lu-Tai Tien, Baoying Zheng, Yi Pang, Rick C S, Lin, Kimberly L Simpson (2011). Dopaminergic neuronal injury in the adult rat brain following neonatal exposure to lipopolysaccharide and the silent neurotoxicity. Brain Behav Immun.

[21] Chung ES, Chung YC, Bok E, Baik HH, Park ES, Park JY (2010). Fluoxetine prevents LPS-induced degeneration of nigral dopaminergic neurons by inhibiting microglia-mediated oxidative stress. Brain RES.

[22] May-Jywan Tsai, Jyh-Fei Liao, Di-You Lin, Ming-Chao Huang, Dann-Ying Liou, Hsin-Chun Yang (2010). Silymarin protects spinal cord and cortical cells against oxidative stress and lipopolysaccharide stimulation. Neurochem Int.

[23] Myers MJ, Shepherd DL, Durr AJ, Stanton DS, Mohamed JS, Hollander JM (2019). The role of SIRT1 in skeletal muscle function and repair of older mice. J Cachexia Sarcopenia Muscle.

[24] Mei ZG, Huang YG, Feng ZT, Luo YN, Yang SB, Du LP (2020). Electroacupuncture ameliorates cerebral ischemia/reperfusion injury by suppressing autophagy via the SIRT1-FOXO1 signaling pathway. Aging (Albany NY)..

[25] Rege S D, Geetha T, Griffin G D, Broderick TL, Babu JR (2014). Neuroprotective effects of resveratrol in alzheimer disease pathology. Front Aging Neuro Sci.

[26] Kumar R, Chaterjee P, Sharma PK, Singh AK, Gupta A, Gill K (2013). Sirtuin1: a promising serum protein marker for early detection of alzheimer disease. PLoS One.

[27] Fang Y, Guo C, Zhang P, Zhao W, Wang S, Xing G (2016). Role of autophagy in methylmercury-induced neurotoxicity in rat primary astrocytes. Arch Toxicol.

[28] Li M, Lindblad J L, Perez E, Bergmann A, Fan Y (2016). Autophagy-independent function of atg1 for apoptosis-induced compensatory proliferation. BMC Biology.

[29] Cerri S, Blandini F (2019). Role of Autophagy in Parkinson’s Disease. Curr Med Chem.

[30] Jang W, Kim H J, Li H, Jo KD, Lee MK, Yang HO (2016). The neuroprotective effect of erythropoietin on rotenone-induced neurotoxicity in SH-SY5Y cells through the induction of autophagy. Mol Neurobiol.

[31] Galluzzi L, Morselli E, Vicencio JM, Kepp O, Joza N, Tajeddine N (2008). Life, death and burial: multifaceted impact of autophagy. Biochem Soc Trans.

[32] Meng L, Ping G, Zhang J (2016). Crosstalk between autophagy and apoptosis: potential and emerging therapeutic targets for cardiac diseases. Int J Mol Sci.

[33] Djavaherimergny M, Maiuri M C, Kroemer G (2010). Cross talk between apoptosis and autophagy by caspase-mediated cleavage of beclin1. Oncogene.

[34] Maiuri M C, Criollo A, Kroemer G (2010). Crosstalk between apoptosis and autophagy within the beclin 1 interactome. EMBO J.

[35] Goodall M L, Fitzwalter B E, Zahedi S, Wu M, Rodriguez D, Mulcahy-Levy JM (2016). The autophagy machinery controls cell death switching between apoptosis and necroptosis. Dev Cell.

[36] Jorge A, Taylor T, Agarwal N, Hamilton DK (2019). Current agents and related therapeutic targets for inflammation after acute traumatic spinal cord injury. World Neurosurg.

[37] Ghosh A, Pal R (2019). Spinal cord injury: Point-of-care biomarkers for better prognosis. J Neurosci Rural Pract.

[38] Mataliotakis G I, Tsirikos AI (2016). Spinal cord trauma: pathophysiology, classification of spinal cord injury syndromes, treatment principles and controversies. Orthopaedics & Trauma.

[39] Hilton BJ, Moulson AJ, Tetzlaff W (2017). Neuroprotection and secondary damage following spinal cord injury: concepts and methods. Neuroscience Letters.

[40] Beattie M S, Farooqui AA, Bresnahan J C (2000). Review of current evidence for apoptosis after spinal cord injury. J Neurotrauma.

[41] Bastianetto S, Ménard C, Quirion R (2015). Neuroprotective action of resveratrol. Biochimica Et Biophysica Acta.

